# Nanosized Thin SnO_2_ Layers Doped with Te and TeO_2_ as Room Temperature Humidity Sensors

**DOI:** 10.3390/s140508950

**Published:** 2014-05-21

**Authors:** Biliana Georgieva, Irena Podolesheva, Georgy Spasov, Jordan Pirov

**Affiliations:** Institute of Optical Materials and Technologies “Acad. J. Malinowski”, Bulgarian Academy of Sciences, Acad. G. Bonchev Str., bl.109, Sofia 1113, Bulgaria; E-Mails: irena18@gmail.com (I.P.); gspassov@iomt.bas.bg (G.S.); jordan_pirov@abv.bg (J.P.)

**Keywords:** SnO_2_, humidity sensors, thin layers, room temperature sensing

## Abstract

In this paper the humidity sensing properties of layers prepared by a new method for obtaining doped tin oxide are studied. Different techniques—SEM, EDS in SEM, TEM, SAED, AES and electrical measurements—are used for detailed characterization of the thin layers. The as-deposited layers are amorphous with great specific area and low density. They are built up of a fine grained matrix, consisting of Sn- and Te-oxides, and a nanosized dispersed phase of Te, Sn and/or SnTe. The chemical composition of both the matrix and the nanosized particles depends on the ratio R_Sn/Te_ and the evaporation conditions. It is shown that as-deposited layers with R_Sn/Te_ ranging from 0.4 to 0.9 exhibit excellent characteristics as humidity sensors operating at room temperature—very high sensitivity, good selectivity, fast response and short recovery period. Ageing tests have shown that the layers possess good long-term stability. Results obtained regarding the type of the water adsorption on the layers' surface help better understand the relation between preparation conditions, structure, composition and humidity sensing properties.

## 1. Introduction

SnO_2_ has been the most studied gas sensing material in the last decades, because of its high sensitivity to numerous reducing gases and to relative humidity (RH [%]) [1]. The first sensor devices were developed on the base of thick SnO_2_ layers. Later on, thin SnO_2_ layers have found an increasing application due to their compatibility with microelectronic technologies. Thin SnO_2_ layers are suitable for measuring the relative humidity, which precise measurement and control are crucial for the industry, agriculture and environmental studies.

The main disadvantages of SnO_2_ sensing layers are their insufficient long-term stability and poor selectivity, *i.e.*, the ability to detect a gas among a mixture of many gases. One possibility to improve these parameters is doping with suitable dopants—metals or other oxides [2].

The sensing properties of tin dioxide depend strongly on the composition and microstructure and ultimately on the method of preparation, therefore the efforts of many researchers are focused on the development of new preparation methods. The aim is the achievement of higher sensitivity and selectivity, fast responses and recovery periods, as well as a long-term stability.

A new method for preparing thin SnO_2_ layers by thermal vacuum co-evaporation of Sn and TeO_2_ was developed at the Institute of Optical Materials and Technologies [3]. It has been found that during the co-evaporation a chemical reaction between the two substances takes place, which results in the formation of a nanosized oxide matrix (SnO_2_) and a finely dispersed phase of Sn, Тe, TeO_2_ and SnТe, depending on the atomic ratio of Sn to Te (R_Sn/Te_) [4,5]:

(1) $$Sn+TeO_{2}\to SnO_{2}+Te$$

(2) $$2Sn+TeO_{2}\to 2\text{SnO}+Te\;(\text{dominant at}\;R_{Sn/Te}>2)$$

(3) $$Te+Sn\to \text{SnTe}\;\left(R_{Sn/Te}>>2\right)$$

The method allows the obtaining of thin Sn-O-Te layers with the following advantages:

- Mixing of the components at an atomic level in desired proportions
- Possibility of obtaining directly doped layers
- Possibility of controllably changing the composition (R_Sn/Te_)
- Possibility of introducing various additional dopants
- Compatibility with conventional microelectronic technologies (possible integration with entire subsystems for data processing).

The aim of this paper is to summarize and to discuss our investigations on the humidity sensing properties of Sn-O-Te layers prepared by thermal vacuum co-evaporation of Sn and TeO_2_. The main parameters of the humidity sensors are studied—sensitivity, selectivity, response time, recovery period and long-term stability. Results concerning the type of the water adsorption on the layers' surface have been obtained, which contribute to the clarification of the detection mechanism of the Sn-O-Te layers, *i.e.*, of the relation between preparation conditions, structure, composition and the resulting sensing properties.

## 2. Experimental

### 2.1. Preparation of the Samples

**Figure 1.**
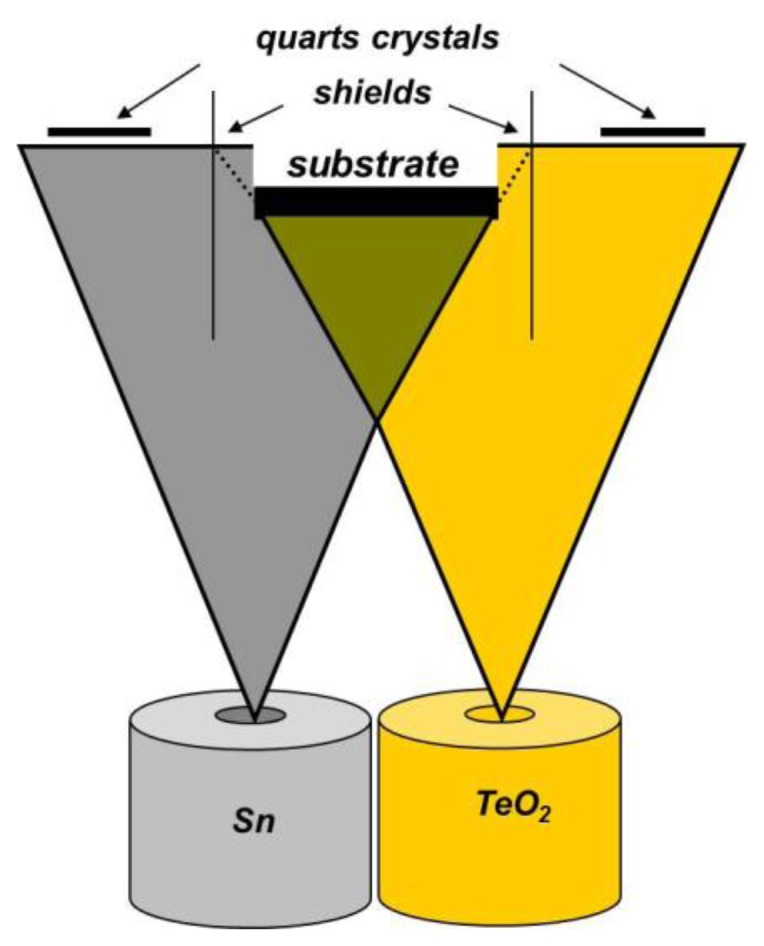
Scheme of the deposition of Sn-O-Te thin layers.

Sn-O-Te layers with thicknesses ranging from 40 to 60 nm were obtained by co-evaporation of Sn and TeO_2_ from independently heated cells, under vacuum better than 10^–4^ Pa (Figure 1). TeO_2_ was evaporated from a Pt-crucible, and Sn from a crucible made of glassy carbon, both of them enclosed, with a small aperture. The condensation rates of both substances were controlled separately during the whole evaporation process using quartz crystal monitors. The chemical composition, *i.e.*, the amount of both substances (the ratio R_Sn/Te_, respectively) and the thickness of the layers were calculated on the basis of the measured evaporation rates using computer programs as described in [3]. Analogous calculation procedures were used for selecting the experimental conditions necessary for obtaining the desired composition and thickness of the samples [3].

In order to ensure high depth homogeneity of the layers' composition, the evaporation was performed onto stationary substrates placed above the evaporation sources. The substrates used were:

- Glass with photolithographically patterned comb-like Cr-electrodes [6] for the electrical measurements.
- Silicon for Energy Dispersed Spectroscopy (EDS) in Scanning Electron Microscopy (SEM) and Auger Electron Spectroscopy (AES).
- Glass with a water-soluble sublayer of polyvinyl alcohol (PVA) for observation in a Transmission Electron Microscope (TEM) and for Selected Area Electron Diffraction (SAED).
- Glass for SEM-profile. The layer with thickness of 1 μm was covered with a conductive carbon layer.

Additional doping with Pt (2–3 at.%) was carried out in a separate vacuum cycle. The doping element was evaporated on the sensing layers using a W basket with a diameter of 0.8 mm.

### 2.2. Characterization of the Sn-O-Te Layers

For characterization of the thin Sn-O-Te layers different techniques were used. The calculated data about the composition, that is about the atomic ratio R_Sn/Te_ and the amount of the dopant (Pt) introduced into the layer, were verified by EDS in SEM and the thickness by a Talystep profilometer (Rank Taylor Hobson Ltd., Leicester, UK).

In order to examine the growth morphology of the layers a SEM observation was carried out on fractured surfaces of samples with 1 μm thickness, covered with a conductive carbon coating. The surface morphology and structure of the sensing layers were studied using TEM and SAED. The samples were prepared by removing the layer from the glass substrate (covered with a water-soluble sublayer of PVA) in water and mounting onto microscope Cu-grids. The thickness of the as-deposited layers (40–60 nm) was suitable for direct observation in TEM.

Auger electron spectroscopy (AES) was used to determine the in-depth distribution of Sn, Te and O (this investigation was made on layers with thickness of 40 nm). The Auger spectra are derivative and obtained by a spectrometer with an energy resolution of ∼0.3% (single-pass CMA and coaxial electron gun). The primary electron energy used was 3 keV and the modulation voltage −4 Vpp. The peak for O (510 eV) and the doublets for Sn and Te were monitored. The profiling was done by sputtering by 3 keV argon ions at a current of 2 μA. The quantification was made through a modified method of the elemental sensitivity factors [7]. SnO_2_ was used as own standard. The intensity of the tellurium doublet was measured from the maximum of the low-energy peak to the minimum of the high-energy peak.

### 2.3. Electrical Measurements

As a measure for the sensitivity of the sensing layers the change in their electrical resistance (R [Ω]) as a function of the relative humidity (RH [%]) was used. The resistance was measured within the interval RH = 30%–100% at temperatures of 25 to 80 °C in a test humidity chamber equipped with temperature and RH controller (TESTO-610 hygrometer, TESTO AG, Lenzkirch Germany). The measurement of R [Ω] of the layers was performed with a multichannel ohmmeter (MCOM), product of National Instruments (Austin, TX, USA). The data acquisition and processing, as well as the control of the ohmmeter, were computerized using LabView software. The experiments concerning the response time and recovery period (kinetic measurements) were performed using saturated solutions of NaOH and (NH_4_)_2_SO_4_ which keep the air humidity within an enclosed space above the solution at 6% and 82%, respectively. The samples were held above the solution and the change of R [Ω] was measured at intervals of 2 s. The hysteresis of the sensor was measured in the humidity chamber at 25 °C. The resistance variation of the sensor was followed with increasing humidity from 30% to 75% and then with decreasing humidity from 75% to 30%, with steps of 10% RH.

## 3. Results and Discussion

### 3.1. Morphology, Structure and In-Depth Distribution of the Elements

**Figure 2.**
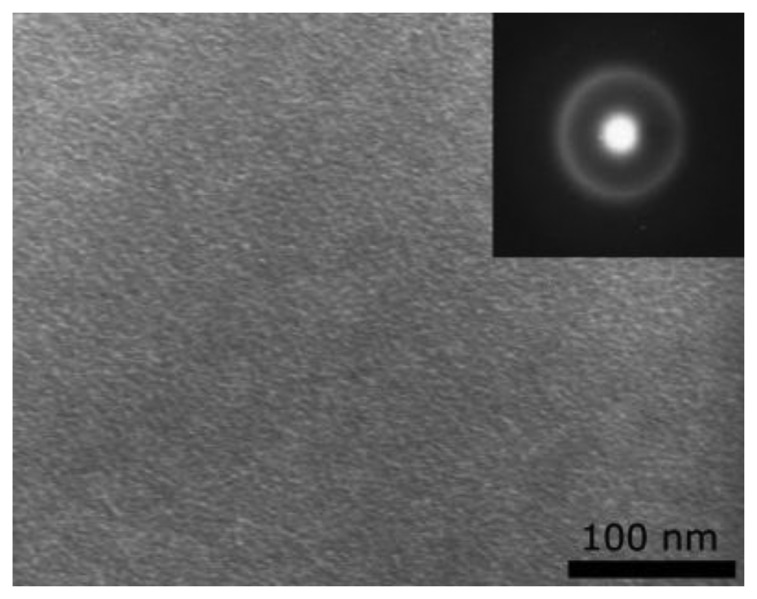
TEM micrograph and SAED pattern of an as-deposited layer with R_Sn/Te_ = 0.7.

**Figure 3.**
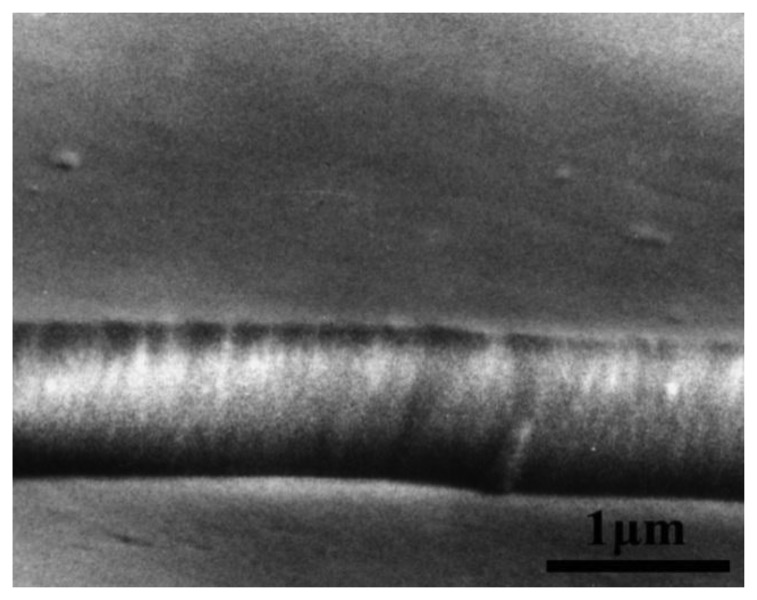
SEM profile of an as-deposited layer with a thickness of 1000 nm.

The layers in the entire range of R_Sn/Te_ from 0.3 to 2.3 are amorphous with a fine granular structure [4,8]. This is illustrated in Figure 2 with the TEM microphotograph and SAED pattern of a layer with R_Sn/Te_ = 0.7. The electron diffraction does not reveal the presence of any crystalline phase. As shown in Figure 3 (reproduced with permission from J. Optoelectr. Adv. Mater., reference 8), besides the granular structure the layers have a columnar structure. Thanks to this, the layers exhibit a great specific area, which is a prerequisite for good gas sensing properties.

**Figure 4.**
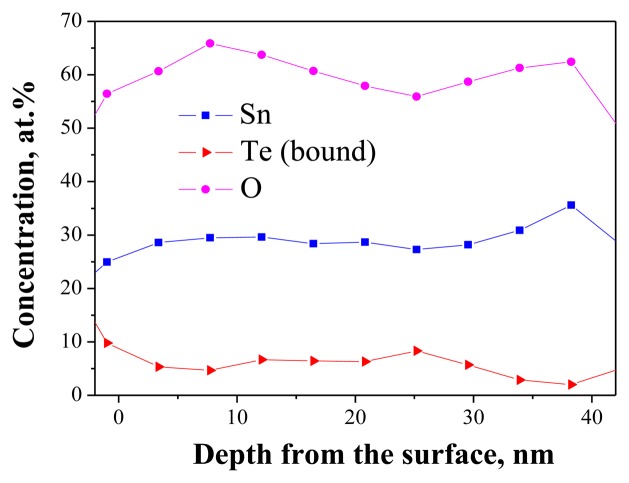
Auger-spectra of in-depth profiling of layers with R_Sn/Te_ = 0.8.

As mentioned above, Auger-electron spectroscopy was used to investigate the homogeneity of the Sn-O-Te layers. However, the direct application of the method is not possible because of the high mobility of the elemental Te present in the layers according to Equation (1). As we have shown in previous investigations on the influence of thermal treatments on the structure and composition of the Sn-O-Te layers, the elemental tellurium starts to diffuse from the bulk and segregates on the layer surface at temperatures above 100 °C [4]. The same process occurs upon electron and ion bombardment and this affects the distribution of Te in the layer. If, however, the mobile elemental Te is removed from the layer prior to the AES analysis, the distribution of the remaining elements—Sn, O and the bonded Te atoms—could be used to assess the homogeneity. Their in-depth distribution should be homogeneous if the original as-deposited layer was homogeneous. The layers used for the analysis were with R_Sn/Te_ = 0.8 (confirmed by EDS in SEM). They contained twenty parts of oxygen, ten parts of tellurium and eight parts of tin (52.6, 26.3 and 21.1 at.%, respectively). The solid state reaction between Sn and TeO_2_ during the co-evaporation resulted in the formation of eight parts of SnO_2_, two parts of TeO_2_ and eight parts of elemental Te. After removal of the elemental Te by the ion beam, the O:Te:Sn-ratio changed to 66.7:6.7:26.7%. As seen in Figure 4, the elements Sn, O and Te are evenly distributed in the depth of the layer. The average plateau concentrations correspond to O:Te:Sn = 61(±1):6.4(±0.4):28.6(±0.3). After correction accounting for the contamination with C (4%), the atomic concentration of O becomes approx. 64%. These results are consistent with those obtained earlier by EDS analysis in SEM at different accelerating voltages which have led to the assumption that the layers are homogeneous [4].

### 3.2. Humidity Sensing Properties

#### 3.2.1. Sensitivity and Selectivity

**Figure 5.**
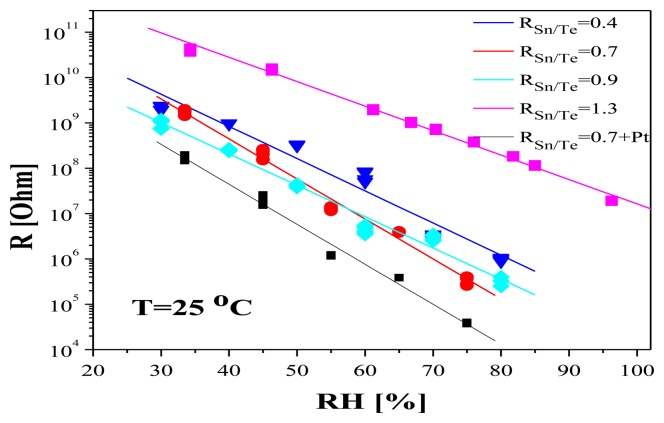
R [Ω] as a function of relative humidity RH [%] for layers with R_Sn/Te_ ranging from 0.4 to 1.3 and for layers with R_Sn/Te_ = 0.7, additionally doped with Pt.

As the Sn-O-Te layers obtained have a nanosized structure, low density and high specific area respectively (the value of ρ = 4 g/cm^3^ is much lower than the respective values for the bulk materials TeO_2_, Te, Sn and SnO_2_—between 5.7 and 7.3 g/cm^3^ [3]) they were tested as humidity sensors. For studying their sensitivity to relative humidity, the change in the electrical resistance R [Ω] with the change of RH [%] was followed. It was found that the as-deposited amorphous layers possess very good humidity sensing properties. Figure 5 presents the response curves of layers with R_Sn/Te_ ranging from 0.4 to 1.3. As can be seen, the electrical resistance of layers with R_Sn/Te_ = 0.4–0.9 decreases with increasing relative environmental humidity spanning 3–4 decades of resistance, the response curve reveals a close exponential relationship between resistance and RH and can be linearized by taking the logarithm of the resistance. Layers with R_Sn/Te_ = 1.3 do not show the same dependence, *i.e.*, only layers with R_Sn/Te_ < 1 possess high sensitivity to humidity.

As we have already mentioned, during the vacuum co-deposition of TeO_2_ and Sn a solid state reaction between Sn and TeO_2_ takes place, resulting in the formation of Sn oxides [4,5,9]. Investigations on Sn-O-Te layers by ^119^Sn conversion electron Mössbauer spectroscopy and XPS (X-ray photoelectron spectroscopy) have unambiguously confirmed that with increasing atomic ratio R_Sn/Te_ (up to 1.3) the amount of TeO_2_ decreases and the amount of Sn-oxides and elemental Te increases. At R_Sn/Te_ < 1 the layers consist of a mixture of Sn oxides (SnO_2_ and some SnO) and TeO_2_, doped with very finely dispersed elemental Te. Obviously, there exists an optimum ratio between the products of the redox reaction (R_Sn/Te_ = 0.7–0.9), which causes the high sensitivity towards humidity at room temperature. It is known that the presence of a second oxide at a level of 2–10 wt.% and of other dopants in SnO_2_-based sensors leads to improved gas response and shifts the maximum of sensitivity to lower operation temperature [10]. The high sensitivity to humidity of layers with R_Sn/Te_ in the range of 0.7–0.9 is probably related also to the non-stoichiometry of SnO_2_, which favors the adsorption of the specific oxygen adsorbate $O_{2}^{-}$ in the temperature range of 25–150 °C [11] and the reaction on the surface:

(4) $$2H_{2}O+O_{2}^{-}+4Sn↔4(Sn-OH)+2e^{-}$$

The release of electrons leads to decreased resistance with increasing humidity. It should be stressed however, that, as noted by other authors too, the mechanism of gas impact on the electrical properties of mixed oxides is much more complicated as compared with homogenous oxides and its detailed understanding needs a more comprehensive study.

The lack of sensitivity to humidity of thermally treated layers is due to significant changes in the in-depth distribution of the layers' components and in the structure (diffusion of the elemental Te to the free surface and formation of a thin Te layer on top of the layer at temperatures about 100 °C, followed by crystallization of the matrix at higher temperatures, *etc.* [4]).

**Figure 6.**
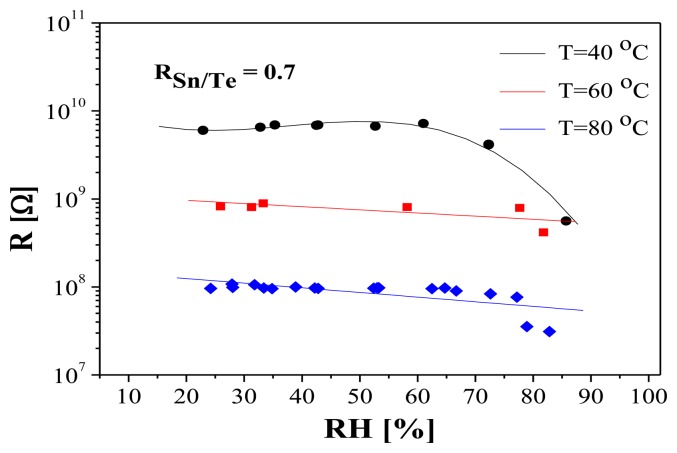
Influence of the temperature on the sensitivity to humidity of amorphous samples with R_Sn/Te_ = 0.7 and thickness of 60 nm.

It is important to note that the high sensitivity to humidity is observed only at room temperature (25 °C). As seen in Figure 6, the humidity does not influence the electrical characteristics of the layers at temperatures over 40 °C. On the base of this result it can be concluded that at temperatures > 40 °C no cross sensitivity to humidity is to be expected if the layers are used as sensors for other gases. However, as seen in Figure 5, at low humidity levels the resistance of the sensing layers is very high (in the order of 10^10^ Ω). One of the ways to overcome this problem is the additional doping with some metals (in our case with Pt). The doping with 2–3 at.% Pt leads to a resistance decrease of one order of magnitude (shown in the same figure) compared with the undoped layers, without any changes in sensitivity [12]. Therefore, a substantial resistance decrease could be attained through optimal doping.

To study the selectivity of the humidity sensitive Sn-O-Te layers their sensitivity towards different vapors (vapors of acids—HNO_3_, H_2_SO_4_, HCl, bases—NH_4_OH and ethanol) at room temperature was checked. No change in the resistance was observed, *i.e.*, the layers exhibit a very high selectivity.

#### 3.2.2. Response Time, Recovery Period and Long Term Stability

**Figure 7.**
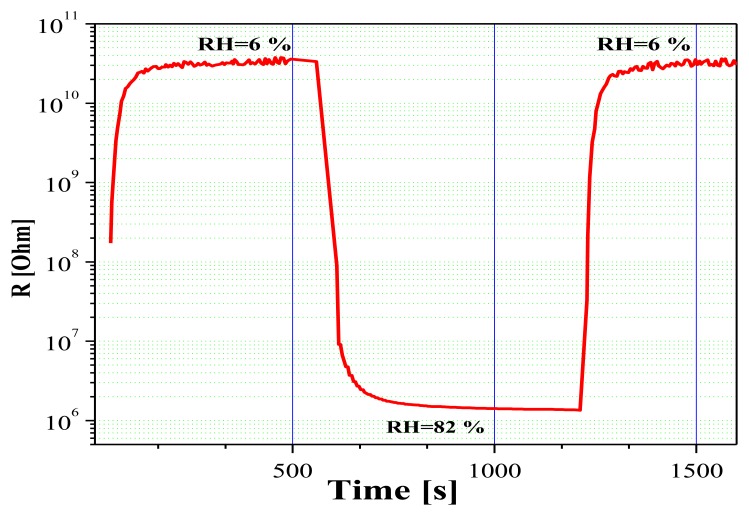
Response time and recovery period of Sn-O-Te layers with R_Sn/Te_ = 0.8.

Further investigations on the sensing properties of Sn-O-Te layers provided information about three of the most important parameters of humidity sensors: response time, recovery period, and long-term stability. Figure 7 shows the sensor response kinetics under step-like changes in RH. As can be seen, the sharp rise in relative humidity (from 6% to 82%) causes a fast response of the sensor, the recovery period being very short as well. This leads to the conclusion that predominantly a physical adsorption of the water molecules on the sensor surface takes place and that the chemisorption plays a minor role. More detailed investigations using Impedance Spectroscopy of layers with R_Sn/Te_ = 0.86 [13] have shown however that at low values of RH [%] the type of the impedance spectra can be explained by prevailing electron conduction through the base sensing material and the adsorbed water on the sensor surface in the stage of chemical adsorption. When humidity increases, ionic conduction also appears as a consequence of the presence of physical adsorption as well. Therefore, the entire conduction mechanism of Sn-O-Te humidity sensor is a combined action of both electron and ionic conduction.

**Figure 8.**
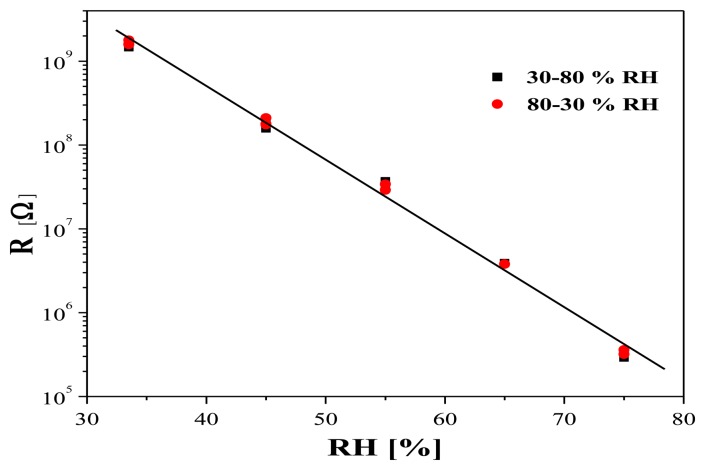
Resistance variation with increasing (from 30% up to 75%) and decreasing (from 75% down to 30%) relative humidity of a layer with R_Sn/Te_ = 0.9.

A typical hysteresis measurement at 25 °C of a layer with R_Sn/Te_ = 0.9 is presented in Figure 8. As it can be seen, the sensor shows no hysteresis, and the resistance variation with increasing humidity from 30% to 75% coincides very well with the respective variation with decreasing humidity from 75% to 30%.

**Figure 9.**
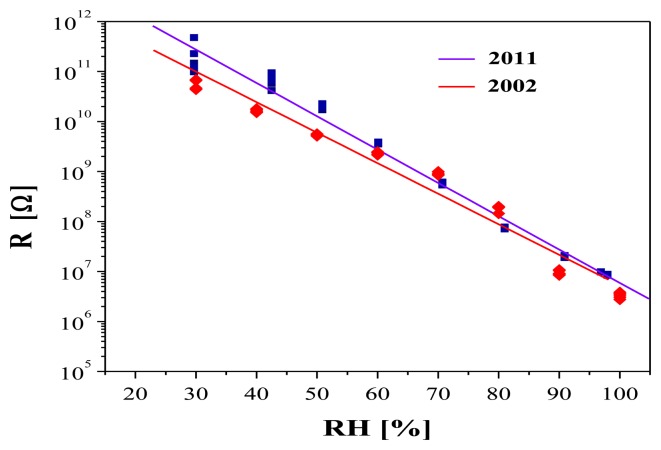
Long-term stability of Sn-O-Te humidity sensors with R_Sn/Te_ = 0.9.

The long term stability is one of the main parameters of the humidity sensors. Results from the ageing tests of layers with R_Sn/Te_ = 0.8 are demonstrated in Figure 9. They indicate that measurements performed over a long period of time (2002–2011) do not show significant differences, *i.e.*, the layers exhibit a very good long-term stability. It is known that additional doping of SnO_2_ layers with other oxides significantly improves the stability [10,14]. Perhaps the presence of TeO_2_ contributes to the high stability of the Sn-O-Te layers.

## 4. Conclusions

The results presented in this paper demonstrate the applicability of the method developed at our Institute for preparing nanosized doped SnO_2_ layers for intended use as humidity sensors. Using different methods the structure and composition of layers, obtained by co-evaporation of TeO_2_ and Sn, their in-depth homogeneity as well as the role of different parameters of the layers for their humidity sensing properties were investigated. It was established that as-deposited amorphous layers with R_Sn/Te_ atomic ratios ranging from 0.7 to 0.9 and thickness < 100 nm, are highly sensitive to water vapors, when operating at room temperature. The sensor response is very fast, the recovery period very short, no hysteresis and cross sensitivity to other gases is observed, and the layers exhibit good long-term stability. The lack of sensitivity to humidity at higher temperatures allows the development of sensors for selective detection of other gases (which usually work at elevated temperatures) in the presence of moisture [15]. A shortcoming of the layers is their high resistance at low humidity levels, but this can be overcome by additional doping with some metals (e.g., with Pt). Studying the sensor response kinetics, the impedance characteristics and the impedance spectra of the humidity sensing layers it was concluded that the entire conduction mechanism is a combined action of both electron and ionic conduction. This is a contribution to the better understanding of the relationship between the method of preparation, structure and sensing properties.

The layer preparation method allows the introduction of finely dispersed dopants in order to obtain materials sensitive to other reduction gases. It is compatible with conventional microelectronics technologies and after optimization the sensors can be integrated on one chip with field-effect transistors or with entire subsystems for data processing.
